# Cross Talk at the Cytoskeleton–Plasma Membrane Interface: Impact on Neuronal Morphology and Functions

**DOI:** 10.3390/ijms21239133

**Published:** 2020-11-30

**Authors:** Rossella Di Giaimo, Eduardo Penna, Amelia Pizzella, Raffaella Cirillo, Carla Perrone-Capano, Marianna Crispino

**Affiliations:** 1 Department of Biology, University of Naples Federico II, 80126 Naples, Italy; eduardo.penna@unina.it (E.P.); amelia.pizzella94@hotmail.it (A.P.); raffaella.cirillo@hotmail.it (R.C.); 2 Department of Pharmacy, University of Naples Federico II, 80131 Naples, Italy; perrone@unina.it; 3 Institute of Genetics and Biophysics “Adriano Buzzati Traverso”, National Research Council (CNR), 80131 Naples, Italy

**Keywords:** cystatin B, cytoskeleton, exosomes, local protein synthesis, plasma membrane, synaptic plasticity

## Abstract

The cytoskeleton and its associated proteins present at the plasma membrane not only determine the cell shape but also modulate important aspects of cell physiology such as intracellular transport including secretory and endocytic pathways. Continuous remodeling of the cell structure and intense communication with extracellular environment heavily depend on interactions between cytoskeletal elements and plasma membrane. This review focuses on the plasma membrane–cytoskeleton interface in neurons, with a special emphasis on the axon and nerve endings. We discuss the interaction between the cytoskeleton and membrane mainly in two emerging topics of neurobiology: (i) production and release of extracellular vesicles and (ii) local synthesis of new proteins at the synapses upon signaling cues. Both of these events contribute to synaptic plasticity. Our review provides new insights into the physiological and pathological significance of the cytoskeleton–membrane interface in the nervous system.

## 1. Axonal Cytoskeleton

Neurons have very specialized and asymmetric shapes. To receive signals from other cells, dendrites of a typical neuron comprise many elongated processes stemming from the cell body. On the other hand, the axon, which conveys signals to neighboring cells through synapses, is often extensively elongated to reach the remote target. The dendrites are multiple in numbers but are merely millimeters long [1]. By contrast, the axons in vertebrates can be as long as a meter and usually start as a single process that is divided into several branches towards its end to form presynaptic terminals or boutons [2]. During development, the neuron initially sprouts multiple immature neurites from the soma, and then, a single neurite grows rapidly to become the axon, whereas other neurites become dendrites [3]. The tip of the growing neurite, named growth cone, has a key role in allowing the axon to elongate toward its target [3]. The unique neuron morphology creates considerable metabolic and mechanical problems to the remote and seemingly feeble subcellular domains such as nerve terminals. Neurons circumvent these problems by use of the unique and intricate network of the axonal cytoskeleton, which allows bidirectional communication between the cell body and presynaptic boutons [2,4]. 

The axonal cytoskeleton has three main components: microtubules, neurofilaments, and actin filaments. Microtubules are cytoskeletal polymers made of 13 protofilaments, forming a cylinder-like structure of about 25 nm in diameter [5]. The protofilament is assembled by heterodimer of α-tubulin and β-tubulin, which are the basic repeating units. The tubulin dimers polymerize in a polarized way. That is, the β-subunit of a tubulin dimer contacts the α-tubulin of the next dimer. Thus, the head-to-tail addition of the dimers leads to a polarized structure in which β-tubulin is exposed in one end (faster-growing “plus” end), whereas the other end exposes α-tubulin (slower-growing “minus” end). Microtubules in all eukaryotic cells play key roles in supporting the cell structure, organelles movement, secretion, and cell divisions. In the axon, microtubules form dense bundles that are parallel to the nerve fibers and uniformly oriented with their plus ends facing the axon terminals [5]. Actin filaments (F-actin), also known as microfilaments, are made by ATP-dependent polymerization of globular actin monomers (G-actin) and are about 8 nm in diameter [4]. The actin filaments are often intimately associated with the plasma membrane in eukaryotic cells. Owing to that, actin filaments not only provide cell rigidity but also actively participate in several critical processes such as cell motility, division, adhesion, and signaling. Neurofilaments, on the other hand, are the intermediate filaments characteristic of neurons. Neurofilaments are about 10 nm in diameter and are heteropolymers composed of three classes of subunits showing high, medium, and low molecular weights. They provide mechanical support to the axon and determine axon diameter [4].

Having axons with such a specialized morphology, neurons are endowed with a special mechanism, named axonal transport, to deliver molecules and organelles to the remote cell periphery. This unique form of transport utilizes microtubules as a polarized railway track on which molecular motors like kinesin and dynein carry cargo towards the plus and the minus ends of microtubule, respectively [6,7]. Although the basic organization of axonal cytoskeleton has been known for a long time, a nanoscale view of axonal cytoskeleton became possible only in the last decade owing to modern technologies such as super-resolution microscopy and live imaging systems. These sophisticated analyses revealed unexpected details of the axonal cytoskeleton. For instance, stochastic optical reconstruction microscopy (STORM) technology visualized a previously unknown actin-based structure in the axon, i.e., ring-like F-actin structures that surround the circumference of axons at regularly spaced intervals (about 180 mm) along the axonal shaft [8,9]. It is worth noticing that this value is below the resolution limit of conventional optical microscopy (250 nm) [10], explaining why these elusive structures were not visualized before. The neighboring actin rings are connected by tetramers of spectrin [9,11]. These periodical cytoskeletal structures are placed in the tight subplasmalemmal area and thus believed to dynamically influence the molecular organization of the plasma membrane. Indeed, it was shown that sodium channels are localized in the axonal membrane in a coordinated way with the underlying actin-spectrin structure [9]. In line with these findings, it was demonstrated that the clustering of voltage-gated sodium channels in the membrane of axon initial segment (AIS) depends on a complex network of cytoskeletal proteins including spectrin, ankyrin-G, and actin [12]. It is noteworthy that the high density of voltage-gated sodium channels in AIS (40–50 times higher than that of the soma and the proximal dendrites) has the crucial physiological implication to make this area the place on the neuron where a decision is made to generate action potential [13]. During development, actin rings first appear in the proximal region of the axon and then propagate toward its distal part [11]. Thus, association of ion channels with the F-actin rings is likely not only to modulate the channel activities but also to arrange the spatial distribution of the channels during development [14,15,16,17]. 

In addition to the subplasmalemmal actin rings, there are also intra-axonal focal “hot spots” of F-actin in the deeper cytoplasm. These sites are highly dynamic with actin undergoing intense polymerization and reorganization, generating long “actin trails”, which may eventually provide actin and its associated proteins for presynaptic boutons and therefore may support physiological processes critical for axonal and synaptic plasticity [18]. Altogether, the experimental data suggest that the organization of actin filaments in the axon is unique in a sense that the stable actin rings mechanically support the plasma membrane and serve as anchoring points for ion channels. In addition, the dynamic actin trails create a flexible cytoskeletal network, essential for the plastic events occurring at the synapse. This combination of properties makes the cytoskeleton strong enough to sustain the long axon but also flexible enough to modify axon shape in response to intrinsic and extrinsic factors. It is noteworthy that the axonal cytoskeleton tightly cooperates with other subcellular structures such as axonal organelles and plasma membrane to make a complex and sophisticated machinery that carries out several physiological processes. The cytoskeleton is utilized for bidirectional trafficking of vesicles involved in secretory and endocytic pathways. In this process, carrier biogenesis is guided by microtubules and F-actin that interact with membranes. This interaction modulates the curvature of the budding vesicles as well as their tension, rigidity, and elasticity during the process of vesicle formation [19]. In support of the idea that vesicle trafficking is linked to actin dynamics, it was shown that F-actin hotspots colocalize with stationary axonal endosomes [18]. Thus, vesicle trafficking seems to be under exquisite multiple controls exerted by the cytoskeleton.

In this regard, it is important to underline that the secretory pathway and membrane recycling mechanisms play crucial physiological roles. For instance, during embryonic development of the nervous system, this membrane trafficking apparatus contributes to neurite outgrowth by serving as a source of the newly synthesized membrane, adhesion molecules, or neurotrophic receptors [20]. In particular, cross talk between the membrane transport and cytoskeletal dynamics mediated by Rab signaling proteins—a family of small GTPases involved in endocytic/exocytic pathways—is involved in neuritogenesis as well as in regeneration of injured axons [20,21,22]. In all eukaryotic cells, secretory and endocytic pathways actively contribute to intercellular communication by transmembrane receptors. Sequestration of these receptors within the endosomal compartments becomes a mechanism to downregulate signal transduction pathways [23]. In this regard, of particular interest to the nervous system is the role played by extracellular vesicles in intercellular communication and in establishing a suitable extracellular environment that has a crucial importance for the development and maintenance of the neural circuitry. 

## 2. Intercellular Communication by Extracellular Vesicles

Extracellular vesicles (EVs) include a group of cell-derived vesicles produced by most cell types which are released into the extracellular space. EVs have functional impact on several physiological processes. While EVs encompass several proteins and nucleic acids, their contents can vary extensively depending on the cell type and the developmental stage. It appears that a highly selective cargo-sorting mechanism determines the precise composition of EVs, which will directly affect their fate and functions [24]. EVs have endosomal origin and are produced and transported inside the cell to be secreted at the plasma membrane. In some polarized cells such as neurons, discrete signals can mediate a localized release of exosomes, a subclass of EVs, by addressing the fusion of multivesicular bodies (MVBs, see below) and the consequent secretion of the intraluminal vesicles to a specific site at the plasma membrane [25]. Upon release, EVs can either act locally or travel in the extracellular space by interacting with extracellular matrix to be eventually internalized by the target cells. Thus, they can comprise short- and long-distance signaling pathways, providing a novel communication mechanism between cells. During neuronal development, such extracellular signals play essential roles in establishing the precise number, position, migration, and function of certain cell types [24,26]. Each step of EV processing, from their intracellular transport and secretion to their internalization into the target cells, is guided by the cytoskeleton and the molecular motors [27]. In particular, the biogenesis and release of EVs are regulated by the “endosomal sorting complex required for transport” (ESCRT). The ESCRT system, which in vertebrates comprises many proteins, represents a complex machinery that sorts vesicles, sequesters cargo, and remodels the membrane [28]. Different components of the ESCRT assemble into functionally different subcomplexes. The ESCRT subcomplex sequesters ubiquitinated cargo, whereas ESCRT-I/II/III directs intralumenal vesicle (ILV) budding. On the other hand, the Vps4 (vacuolar protein sorting-associated protein) complex ensures final membrane scission and/or ESCRT recycling [28,29]. ESCRT promotes bending of endosomal membranes to generate MVBs and plays essential roles not only in releasing retroviruses and extracellular vesicles but also in pruning neuronal processes and in repairing both plasma and nuclear membranes. The latter process is essential in regulating the quality of nuclear pore components [30,31,32]. All the processes controlled by ESCRT require membrane remodeling and cytoskeletal restructuring. Interestingly, modifications of ESCRT or of the microtubular system can have pathological effects. In particular, ESCRT-III comprises eleven subunits designated CHMPs (charged multivesicular body proteins) that are particularly important for membrane scission. Indeed, loss-of-function mutation in the ESCRT-III member CHMP1A impairs EV biogenesis by reducing intraluminal vesicles within MVBs and therefore disrupts secretion of a distinctive Sonic HedgeHog-positive EV subtype [33]. Interestingly, this mutation has a striking effect on the nervous system, causing autosomal recessive microcephaly [33].

**Figure 1:**
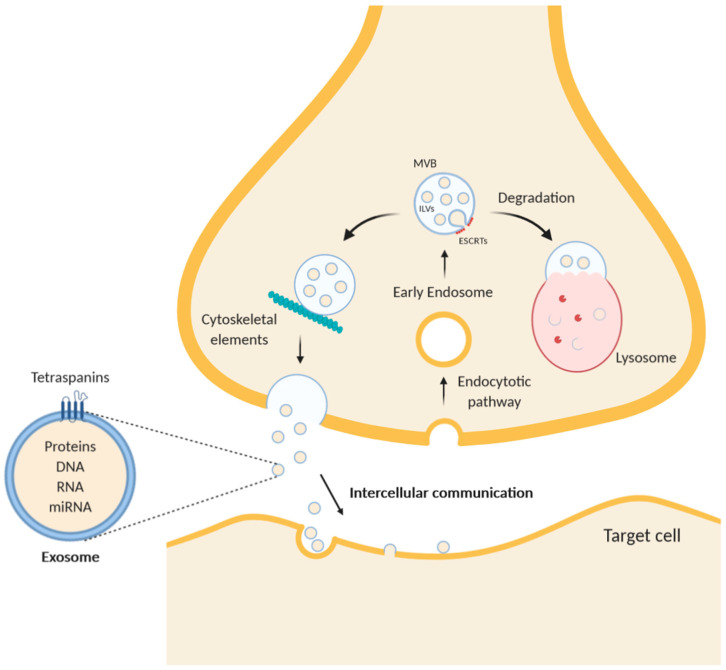
Schematic diagram of exosome trafficking in a neuron. While multivesicular bodies (MVBs) undergo a maturing process, the composition of the intraluminal proteins is gradually changed. The endosomal sorting complex required for transport (ESCRT) remodels the inner membrane of MVB to generate the intraluminal vesicles (ILVs), which represent exosomes inside the MVBs. After intracellular translocation mediated by cytoskeleton elements, such as F-actin, and microtubules, MVBs merge with the plasma membrane and undergo exocytosis. Alternatively, MVBs can fuse with lysosomes to degrade themselves. Exosomes contain in their lumen free proteins, RNA, microRNA, and DNA and are characterized by transmembrane proteins of the tetraspanins family.

Most of the EVs are defined as either exosomes or microvesicles, based on their size, composition, and origin [24]. Exosomes, the smallest class of extracellular vesicles, are generated as ILV through inward budding of the membrane of MVB, an intracellular endosomal organelle typically with a diameter of 250–1000 nm [34,35]. The MVB has two potential fates: it can fuse with lysosomes to degrade its content or fuse with the plasma membrane, releasing its ILV content as exosomes into the extracellular space (Figure 1). This process is different from conventional exocytosis in a sense that the extruded material is still enclosed by vesicular membranes with a typical lipid composition resembling raft microdomains [36,37]. MVBs fusion with the plasma membrane is facilitated and controlled by Rab proteins, which participate virtually in all the processes related to vesicle transport within cells [38,39]. The mechanistic detail of their action is largely unknown, but the involvement of specific Rab proteins has been demonstrated in endosome motility and exosome secretion [40]. Besides Rab proteins, exosome secretion also depends on other molecules involved in the translocation of MVB to the plasma membrane via cytoskeleton [25]. For example, Kif2c is a neuron-specific motor protein belonging to the kinesin superfamily, which is specifically expressed in dendrites and in the cell body. Interestingly, it was demonstrated to be associated with MVB. Since Kif2c moves directionally toward the plus-end of microtubules, it is a good candidate to translocate MVB to the neuronal plasma membrane in a polarized way [41]. Besides microtubules, actin filaments also participate in MVB transport toward plasma membrane. Indeed, it has been shown that Alix (programmed cell death 6-interacting protein), a protein involved in endocytic membrane trafficking, plays a role in connecting cortical F-actin and MVB [42]. In conclusion, the continuous transport of MVBs to the plasma membrane is performed via their interaction with cytoskeletal elements such as microtubules and F-actin [27].

Once secreted, exosomes exert multiple functional effects on the extracellular environment. An emerging concept suggests that exosomes are novel cell-to-cell communication mediators playing important roles in diverse physiological and pathological conditions [43,44]. In the brain, they are involved in numerous processes including synaptic plasticity, neuronal stress response, and neurogenesis [44]. In this context, it should be remembered that development of the mammalian brain requires finely orchestrated events spanning from neurogenesis, cell migration, axonal projections, and synaptogenesis. All these steps imply exquisite control of the cytoskeletal architecture and remodeling [45,46,47]. In the cortex, neurons reach their final destinations to be integrated into the six different horizontal layers, following not only their own genetic program but also signals coming from the surrounding cells and from the cerebrospinal fluid [48]. In this framework, cell communication via exosomes might play a significant role. In support of this hypothesis, the neurogenic role of exosomes has been demonstrated in an elegant work in which exosomes injected into the lateral ventricles of mouse brain stimulated hippocampal neurogenesis [49]. In addition, exosomes released by human primary neural cultures are able to rescue the deficits in proliferation, differentiation, and synaptogenesis exhibited by MECP2-deficient neuronal cultures, an in vitro model of the neurodevelopmental disorder Rett syndrome. Strikingly, endogenous exosomes released by MECP2-deficient neuronal cultures lack this capability. Altogether, these results indicate that, in physiological conditions, exosomes carry signaling information required to establish correct neural connectivity and synaptic strength. Dysregulation of the exosome processing has been implicated in neurodevelopmental disorders as well as psychiatric conditions and neurodegenerative disorders [49,50,51,52,53]. For instance, exosomes from the brain of Alzheimer’s disease (AD) patients not only contain amyloid-beta oligomers but also, in vitro, are able to spread these oligomers from neuron to neuron [53].

The finding that all aspects of exosomal trafficking and extrusion are exquisitely controlled by cytoskeletal elements moves our focus onto the molecular events near the plasma membrane. What molecules are packed in exosomes, and how are they extruded to the outside of the plasma membrane? What is the impact on the extracellular matrix organization? One clue may be found in a recent study utilizing a 3D human neurogenic model of EPM1, a rare form of epilepsy [54]. EPM1 is a neurodegenerative disorder accounting for the highest incidence of progressive myoclonus epilepsies worldwide [55]. Mutations in cystatin B (CSTB) are the primary genetic cause of EPM1 [56]. Interestingly, many CSTB partners are involved in cytoskeletal functions [57,58,59], raising the possibility that impairment of such a cytoskeletal interaction displayed by mutated CSTB may underlie the pathological condition. Moreover, low levels of functional CSTB can lead to decreased recruitment of interneurons, suggesting that CSTB may play a novel role by determining the composition of the extracellular environment [54]. In line with these findings, it has been demonstrated that CSTB is secreted by rat cerebral cortex synaptosomes, an in vitro model of the synaptic region [60]. Altogether, these results raise intriguing questions on how CSTB arrives at the cytoskeleton–plasma membrane interface to be secreted by nerve endings possibly as exosomes cargo. Answers to these questions will shed a new light into the role of CSTB in cell-to-cell cross talk. Interestingly, CSTB was also shown to be involved in synaptic plasticity in mammalian brains. Indeed, it was demonstrated to be locally synthesized in rodent brain synaptosomes and secreted by them in a depolarization-dependent way [60]. Since impairment of the CSTB functions is known to lead to neurodegenerative disorders, it is possible that failure of the finely regulated intracellular cytoskeleton–membrane cross talk may contribute to the altered synaptic plasticity characterizing several neuropathologies.

Biogenesis of extracellular vesicles and their trafficking require exquisite cross talk between the cytoskeleton and plasma membrane, involving multiple molecular components. In a neuron, packing various proteins and nucleic acids in exosomes and carrying them over to a remote subcellular zone to release them into the extracellular territory would serve as another mechanism by which a neuronal biochemical signal is dispatched to the cell territory to affect other cells. Such signaling routes would eventually help to establish specific cell-to-cell connection and to configure neural circuitry. The fine-tuning of the neural circuitry depends on modulation of synaptic contacts in response to stimuli, namely synaptic plasticity. We now move our attention to another molecular mechanism underlying synaptic plasticity, the local synthesis of proteins in the axon and presynaptic terminals. Here again, local cytoskeleton is implicated, and its fine regulation plays a pivotal role.

## 3. Local Protein Synthesis as Molecular Mechanism of Synaptic Plasticity

In a highly polarized neuron, synaptic terminals are often located remotely from the soma. Each of these terminals establishes precise contact with other cells, allowing the formation of the articulate neuronal network that is responsible for brain activity. Remarkably, the brain is able to change the cytoarchitecture of its neuronal circuits in response to various stimuli. The synaptic plasticity, which is indeed defined as the ability of the brain to adapt and modulate synaptic strength and connections, plays a crucial role in both physiology and pathology of the nervous system [61]. An important contribution to synaptic plasticity is made by the local system of protein synthesis present in synaptic areas. Indeed, a growing body of evidence suggests that proteins may be synthesized not only in the soma but also in the synaptic regions in response to local signals [2,62,63,64,65,66,67]. This on-site and on-demand synthesis of proteins allows mRNA translation to take place independently from cell body, making the individual synapse able to quickly respond to the stimuli. The local system of translation can also discern different synapses on the same axon [2]. Supporting the physiological relevance of local protein at synapses, studies in the last few decades have demonstrated the involvement of synaptic translation in various experimental models of brain plasticity, while its deregulation is linked to aging and to several neuropathologies [68,69,70,71,72,73,74].

What is most remarkable is that the synaptic system of protein synthesis has discrete locations in the axon and presynaptic terminals. Indeed, in myelinated axons of goldfish and mammals, ribosomes are discretely distributed in a small area underneath the plasma membrane, close to myelin sheath named “periaxoplasmic plaques” [75,76]. Whether the ribosomes and RNAs for local translation originate from the neuron itself or from the periaxonal glial cells [63,65,77,78], this was the first indication that the plasma membrane and its associated structures may serve as a scaffold for protein synthesis in the axonal and synaptic territory. This hypothesis was confirmed years later when it was shown that ribosomes in the growth cones are physically associated with the netrin-1 receptor DCC (Deleted in Colorectal Carcinoma), a transmembrane protein. When netrin binds to this receptors, the quiescent ribosomes are released from the membrane and start to translate mRNAs possibly using the cytoskeleton as a physical support [79]. This elegant work suggested a model where a transmembrane protein mediates the interplay between extracellular signal and the synaptic system of protein synthesis. Notably, this hypothesis represents a novel molecular mechanism by which the nerve terminals respond to external stimuli. The receptor-bound ribosomes, confined in the plasma membrane but moving to the cytoskeleton upon signaling cue to start producing new proteins locally, may serve as a molecular switch that transduces the external signal [79].

The physical association of ribosomes and polysomes with the cytoskeleton has long been known, and it was hypothesized that this ribosomes-cytoskeleton binding is a strategy of the cell to sort out newly synthesized proteins and to differentially distribute them to the specific subcellular compartments in response to physiological stimuli [80]. A similar conclusion was drawn by a work performed in neurons of the worm *Caenorhabditis elegans*, which demonstrated that ribosomes present at the presynaptic terminals are aggregated into clusters resembling the periaxoplasmic plaques of the vertebrate axons [76,81]. In particular, it has been reported that subcellular localization of ribosomes inside the neuron is dependent upon microtubules and UNC-16, a protein working as an adaptor between kinesin and its cargos [81]. Thus, it appears that the local system of protein synthesis owes a great deal to the cytoskeleton for its distribution and activation.

The importance of cytoskeletal structures dynamically modulated near the plasma membrane is highlighted by their presence also in the growth cones, which respond to external stimuli by making new proteins locally [82,83]. In the developing neuron, the axon prolongs itself to a distance to reach its target. This is made possible because growth cones detect extracellular signals distributed along the way. Thus, different cues expressed in specific regions of the embryonic central nervous system attract or repel growth cones carrying the appropriate membrane receptors. The growing axons can respond to these cues independently from the cell body, mainly relying on the local system of protein synthesis [84]. Interestingly, an attractive cue, netrin-1, binds to its receptor on the growth cone membrane and thereby triggers local translation of β-actin mRNA. The newly synthesized β-actin, asymmetrically concentrated on the side of the growth cone closest to the netrin-1 source, provides nucleation sites for actin polymerization. The directional polymerization of F-actin turns the growth cone towards the cue [85]. On the other hand, the repulsive guidance cue, slit-2, causes local translation of cofilin, an actin-depolymerizing protein. Local increase of cofilin leads to disassembly of the cytoskeleton through severing of actin filaments and consequently to space-specific collapse of the growth cone [86]. Altogether, these data indicate that extracellular cues, binding to their specific membrane receptors on the growth cones, evoke local synthesis of appropriate cytoskeletal proteins that promote either assembly or disassembly of cytoskeletal components and therefore decide the turning direction of the growth cones [87]. This sophisticated interaction between membrane and the cytoskeleton, with the molecular supply of synaptic protein synthesis, is an ingenious mechanism to control the elongation of the axon toward its target [88], which is a fundamental process in the development of the nervous system.

## 4. Cytoskeleton Dynamics in Synaptic Plasticity

Roles played by the actin cytoskeleton in synaptic plasticity are not restricted to the growth cones of developing neurons. In mature neurons, the actin dynamics at the synapse have a crucial role in controlling exocytosis and recycling (endocytosis) of synaptic vesicles [89,90]. Without this fundamental process that requires nearly perpetual local remodeling of the plasma membrane and the subjacent cytoskeleton, neuronal activity would not hold. Recently, an unexpected role of actin in axonal vesicle trafficking was reported. The axonal movement of conventional transport of vesicles along the axons is a microtubules-based movement. Intriguingly, it was shown that the trafficking of the recycling presynaptic vesicles retrieved after exocytosis depend on actin filaments instead of the microtubule system. This actin-based trafficking is regulated by protein kinase A (PKA) and nitric oxide (NO) [91]. The authors hypothesized that, in potentiated synapses, the retrograde signaling mediated by NO leads to inhibition of actin elongation and recycling vesicle transport. As a result, vesicles are stalled at presynaptic endings involved in potentiation. Thus, actin dynamics at the synapse play a critical role in supporting synaptic plasticity [91].

**Figure 2:**
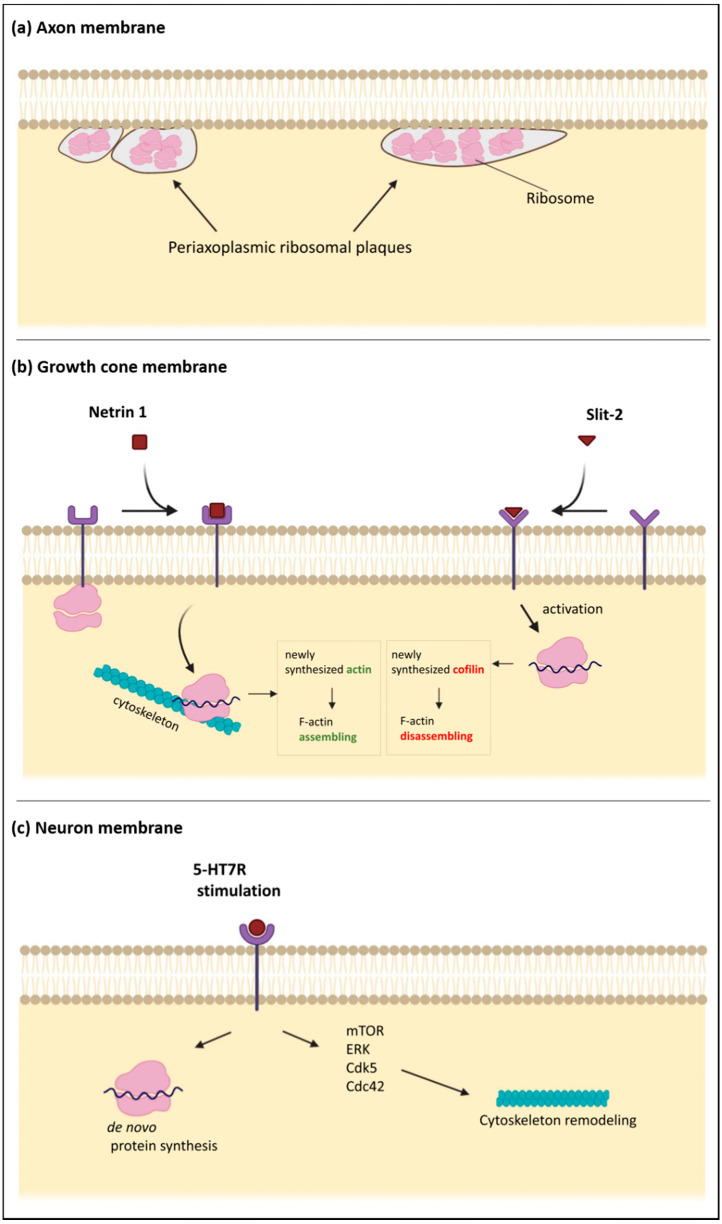
Schematic diagrams of the subplasmalemmal regions in specialized neuronal compartments. (**a**) the axonal plasma membrane is associated with “periaxoplasmic plaques”, which contain ribosomes and therefore serve as a center for intra-axonal protein synthesis. (**b**) The plasma membrane of the growth cones contains receptors for various guiding cues. When netrin-1 receptor DCC (Deleted in Colorectal Carcinoma) is bound by netrin, the sequestered ribosomes are released from the membrane and move to the unspecified cytoskeleton, which may serve as a scaffold for protein synthesis. Translation of β-actin mRNA increases local concentration of β-actin, which leads to enhanced F-actin polymerization. Binding of a repulsive guidance cue slit-2 has the opposite effect because of the increased local translation of cofilin, an actin-binding protein. (**c**) Stimulation of the serotonin receptor 7 (5-HT7R), localized in the neuronal plasma membrane, promotes de novo protein synthesis and activation of several signaling pathways such as ERK, Cdk5, Cdc42, and mTOR. These intracellular pathways converge to promote reorganization of the neuronal cytoskeleton, including microtubules and actin filaments.

Another example of extracellular signals affecting the development of the nervous system by targeting the cytoskeleton is found in the stimulating effect of serotonin receptor 7 (5-HT7R) on neurite outgrowth, synaptogenesis, and synaptic plasticity [92,93,94]. In recent years, 5-HT7R has gained increasing attention for its central role in the plasticity of the nervous system, particularly in the establishment and remodeling of neuronal cytoarchitecture during development as well as in adults. Accordingly, its dysfunctions are linked to neuropsychiatric and neurodevelopmental diseases [95,96] In rodent neuronal primary cultures, stimulation of 5-HT7R promotes neurite elongation, and this effect depends on de novo protein synthesis and the activation of several signaling pathways, such as ERK, Cdk5, the RhoGTPase, Cdc42, and mTOR. These signaling systems converge to promote reorganization of the neuronal cytoskeleton by modulating selected proteins such as microtubule-associated proteins and the actin-binding protein cofilin [92,93] (Figure 2).

In summary, it is possible to hypothesize that plastic events, such as modulation of neuronal shape and connectivity during CNS development and maintenance, rely on the interaction between the plasma membrane and the subjacent cytoskeleton. In the given context, another interesting aspect to consider is related to trafficking of the proteins locally synthesized in the presynaptic terminals. Some of these newly synthesized proteins are secretory proteins such as cystatin B, amyloid precursor protein (APP), or transmembrane proteins such as ion channels [54,60,71,97]. Therefore, they require the presence of the machinery involved in the secretory pathway at the synaptic level. The secretory pathway, which is the main source of the plasma membrane, typically includes the endoplasmic reticulum (ER), Golgi complex, and post-Golgi intermediates [98]. While the presence of a satellite secretory pathway, often including the ER and Golgi systems, has been demonstrated in dendrites [99,100], the analogous apparatus in the axon has been intermittently reported [101,102,103]. In this regard, it is particularly meaningful that a membrane protein locally synthesized in the axon was demonstrated to be actually functional. That is, potassium channel subunits being synthetized in the squid giant axon are also locally folded and tetramerized to be inserted into the plasma membrane and function as an ion channel, raising the possibility that an axoplasmic endomembrane system exists and is at work [97]. In support of the idea that the cytoskeleton is involved in this process, it has been demonstrated that ion channels directly or indirectly bind to actin filaments [12,14,104].

It is noteworthy that synaptic plasticity depends on the fine balance between synthesis of new proteins and degradation of aged proteins [105]. In this context, it is important to mention the autophagy machinery, responsible for degradation of intracellular material through lysosomes [106]. Autophagy is a system to control cellular protein quality, a process that is particularly relevant in neurons to eliminate toxic materials and to maintain axonal homeostasis. Indeed, in neurons, most of the autophagosomes are built up in the axon and are transported back to the cell body to be degraded in lysosomes. This axonal retrograde transport is microtubule-dependent and uses dynein as a motor protein [107]. A series of proteins are involved in the complex autophagosome biogenesis that requires elongation and sealing of isolation membrane [108]. Recently, ESCRT-III member CHMP2A was identified as a key regulator of the sealing step of autophagosome formation [109]. The importance of autophagy in neuronal activity is demonstrated by the fact that dysregulation of the autophagy machinery is linked to axonal degeneration and neurodegenerative diseases [110]. Accordingly, in a mouse model of Alzheimer’s disease (AD), alterations in microtubule network and molecular motors resulted in defective axonal transport, axonal autophagic vesicle accumulation, and dystrophy of neurites [111]. In addition, the abnormal accumulation of autophagosomes in presynaptic terminals leads to their morphological disruption, an early feature of AD before neuronal loss. Indeed, the appropriate degradation of synaptic components by autophagy significantly contributes to synapse remodeling [105]. Interestingly, it was demonstrated that synaptic localization of autophagosome depends on the kinesin KIF1A/UNC-104 [112]. Moreover, defects in autophagy are accompanied by altered localization of F-actin and synaptic vesicles to presynaptic sites, indicating a tight link between the autophagy pathway and the cytoskeleton in synaptic areas [112]. Notably, the secretory and autophagy pathways are interconnected processes and this interaction plays a crucial role in membrane remodeling since components of the secretory pathway are a source of membrane for autophagy [113]. Accordingly, it was demonstrated that dysfunctions in the endosomal-lysosomal system alter the production and secretion of exosomes, while the exosome biogenesis can affect the endosomal–lysosomal system [114]. Another interesting aspect to consider in the context of synaptic plasticity is the synaptic localization of mitochondria to support the high energy requests of this area. To differentially distribute mitochondria in the different neuronal compartments, several neuronal cytoskeleton elements work in integrated way [115]. In addition, some cytoskeleton proteins are implicated in mitophagy, a process that is particularly relevant in neuronal cells requiring sophisticated mitochondrial quality control mechanisms [115]. In conclusion, the crosstalk between mitochondria and the neuronal cytoskeleton proteins is of great relevance in the physiology of neuron and synapse.

## 5. Cytoskeleton Dysfunctions and Neurological Disorders

Given the role of the cytoskeleton in neuronal development and maintenance, it is not surprising that its derangement is tightly associated to several pathological conditions of the CNS [116]. One example is represented by Periventricular Heterotopia (PH) disorders, a group of neurodevelopmental diseases displaying impairment in cortical layers organization due to altered migration of subpopulation of new-born neurons. In PH patients, a mutation of the Endothelin-converting enzyme-2 (*ECE2*) gene has been described. Interestingly, ECE2 influences both organization of actin filaments and the stability of microtubules [117]. In a different type of heterotopia involving downregulation of the *RhoA* gene, actin filaments and microtubules are destabilized in radial glia as the G/F-actin ratio is increased. The altered radial glia scaffold leads to improper neuronal migration [118].

In general, the alteration of cytoskeleton observed in diverse neurological disorders refers to the anomaly in the three aforementioned classes of cytoskeletal proteins in the brain. One key organizer of neuronal actin, microtubule, and neurofilament dynamics is Cdk5, a member of the cyclin-dependent kinase superfamily. Cdk5 plays a critical role in various neuronal processes during development as well as in the adult brain. Thus, it is not surprising that its deregulation is implicated in numerous brain disorders, including Alzheimer’s disease, Parkinson’s disease, Huntington’s disease, attention-deficit hyperactivity disorder, epilepsy, schizophrenia, and ischemic stroke, all characterized by alteration of the neuronal cytoskeleton [119,120].

Dysfunctions of the microtubule system are tightly associated to neurodegeneration. Indeed, neurodegenerative disorders are often accompanied by altered expression of tubulins, microtubule-associated proteins (MAPs), and microtubule modifying enzymes [121]. Accordingly, in vitro analyses and preclinical studies have demonstrated beneficial effects of agents modulating microtubule stability or affecting posttranslational modification of tubulins. These data suggest microtubules as a possible target for therapeutic treatment of neurodegenerative diseases [121,122]. On the other hand, altered actin dynamics contribute to pathogenesis of neurodegenerative diseases. Indeed, in Alzheimer’s disease, an anomaly was found in the actin-binding protein cofilin, suggesting that impaired actin remodeling may also have a pathological consequence [123,124]. Moreover, alteration of neurofilament proteins has been shown to be involved in neuropathology. Indeed, changes in the expression levels and posttranslational modification of NF subunits are linked to the neuropsychiatric conditions characterized by altered synaptic plasticity [125]. In addition, increased levels of the neurofilament light subunit (NFL) have been detected in cerebral spinal fluid and serum of the patients with neurodegenerative diseases [126]. Thus, NFL can be used as an important biomarker for early diagnoses of these diseases [127].

The curious presence of NFL in the extracellular fluid suggests a close interaction between the NFL and plasma membrane and calls for future investigations on this issue. Also, secretion of the microtubule-associate tau protein from the neuron depends on the interaction between cytoskeleton and plasma membrane [128]. Indeed, tau, besides its crucial role in regulating proper cytoskeletal organization and trafficking of microtubules, is constitutively and physiologically secreted outside the cells, although many aspects of its secretion remain unknown [128]. In an AD brain, hyperphosphorylated tau proteins create intracellular aggregates, named neurofibrillary tangles, that are one of the neuropathological hallmarks of the disease [129]. Tau pathology can diffuse in the brain using a prion-like mechanism. Indeed, tau aggregates can transfer from pathological to healthy neurons, inducing in them misfolding and aggregation of tau molecules. Pathological tau can be secreted by the neuron using different pathways including exosome release and direct secretion through plasma membrane [128]. In addition, tau can be also transferred directly from cell to cell by the tunneling nanotubes (TNTs). TNTs are novel F-actin-based membranous channels which represent a recently identified cell-to-cell communication system based on cross talk between cytoskeleton and plasma membrane [130].

## 6. Conclusions

The data reviewed here highlighted how cytoskeleton dynamics and its interaction with plasma membrane serve as a general mechanism by which neurons respond to the given challenges by trafficking exosomes or launching local protein synthesis in the nerve endings leading to synaptic plasticity. It is noteworthy that a commonplace subcellular entity such as the cytoskeleton can be utilized for the two different processes discussed here. For the exosomal signaling pathway, cytoskeletal elements interact with both plasma membrane and vesicular membrane to enable intracellular signaling, allowing also communication of neuron with other cells in the extracellular environment. In the tight space of nerve terminals, a local system of protein synthesis is shuttling between the plasma membrane and cytoskeleton to repress or activate the translational activity of the ribosomes present in the synaptic regions. Thus, the cytoskeleton subjacent to the plasma membrane does not merely play a structural role mechanically strengthening the plasma membrane, but it changes itself to achieve synaptic plasticity by interacting with the vital systems present at the plasma membrane.

## Abbreviations

AD	Alzheimer’s disease
AIS	Axon Initial Segment
CSTB	Cystatin B
ER	Endoplasmic Reticulum
ESCRT	Endosomal Sorting Complex Required for Transport
EV	Extracellular Vesicle
ILV	IntraLumenal Vesicles
MVB	MultiVesicular Body
5-HT7R	Serotonin receptor 7
